# Cross talk between *β* subunits, intracellular Ca^2+^ signaling, and SNAREs in the modulation of Ca_V_2.1 channel steady‐state inactivation

**DOI:** 10.14814/phy2.13557

**Published:** 2018-01-29

**Authors:** Selma Angèlica Serra, Gemma G. Gené, Xabier Elorza‐Vidal, José M. Fernández‐Fernández

**Affiliations:** ^1^ Laboratori de Fisiologia Molecular Departament de Ciències Experimentals i de la Salut Universitat Pompeu Fabra Barcelona Spain

**Keywords:** Ca^2+^‐calmodulin, Ca_V_2.1 domains for SNARE‐mediated modulation, Ca_V_2.1 steady‐state inactivation, Ca_V_*β* subunits, presynaptic voltage‐gated Ca_V_2.1 channels, syntaxin‐1A

## Abstract

Modulation of Ca_V_2.1 channel activity plays a key role in interneuronal communication and synaptic plasticity. SNAREs interact with a specific *synprint* site at the second intracellular loop (LII‐III) of the Ca_V_2.1 pore‐forming *α*_1A_ subunit to optimize neurotransmitter release from presynaptic terminals by allowing secretory vesicles docking near the Ca^2+^ entry pathway, and by modulating the voltage dependence of channel steady‐state inactivation. Ca^2+^ influx through Ca_V_2.1 also promotes channel inactivation. This process seems to involve Ca^2+^‐calmodulin interaction with two adjacent sites in the *α*_1A_ carboxyl tail (C‐tail) (the IQ‐like motif and the Calmodulin‐Binding Domain (CBD) site), and contributes to long‐term potentiation and spatial learning and memory. Besides, binding of regulatory *β* subunits to the α interaction domain (AID) at the first intracellular loop (LI‐II) of *α*_1A_ determines the degree of channel inactivation by both voltage and Ca^2+^. Here, we explore the cross talk between *β* subunits, Ca^2+^, and syntaxin‐1A‐modulated Ca_V_2.1 inactivation, highlighting the *α*_1A_ domains involved in such process. *β*_3_‐containing Ca_V_2.1 channels show syntaxin‐1A‐modulated but no Ca^2+^‐dependent steady‐state inactivation. Conversely, *β*_2a_‐containing Ca_V_2.1 channels show Ca^2+^‐dependent but not syntaxin‐1A‐modulated steady‐state inactivation. A LI‐II deletion confers Ca^2+^‐dependent inactivation and prevents modulation by syntaxin‐1A in *β*_3_‐containing Ca_V_2.1 channels. Mutation of the IQ‐like motif, unlike CBD deletion, abolishes Ca^2+^‐dependent inactivation and confers modulation by syntaxin‐1A in *β*_2a_‐containing Ca_V_2.1 channels. Altogether, these results suggest that LI‐II structural modifications determine the regulation of Ca_V_2.1 steady‐state inactivation either by Ca^2+^ or by SNAREs but not by both.

**Key points summary**

- The functional interaction between presynaptic voltage‐gated Ca^2+^ channels (Ca_V_2.x) and soluble N‐ethylmaleimide‐sensitive factor attachment protein receptor (SNARE) proteins of the secretory machinery, optimizes neurotransmitter‐mediated interneuronal communication.
- Alteration of such functional interaction has clinical relevance in the context of neurological disorders such as ataxia and migraine.
- Regardless of the important anchoring function of a specific Ca_V_2.x region (the *synprint* site) in the Ca_V_2.x‐SNAREs interaction, the involvement of other channel domains has been proposed.
- By combining heterologous expression in HEK 293 cells, whole‐cell patch‐clamp and site‐directed mutagenesis, we show that Ca_V_2.1‐SNAREs functional interaction entails Ca_V_2.1 molecular determinants beyond the *synprint* site, including the first intracellular loop and the carboxyl tail, and their physical interaction with regulatory *β* subunits and the Ca^2+^‐calmodulin complex, respectively.
- Altogether help us better understand the molecular machinery that initiates and regulates vesicles fusion with the presynaptic plasma membrane to trigger chemical neurotransmission.

## Introduction

**Figure 1:**
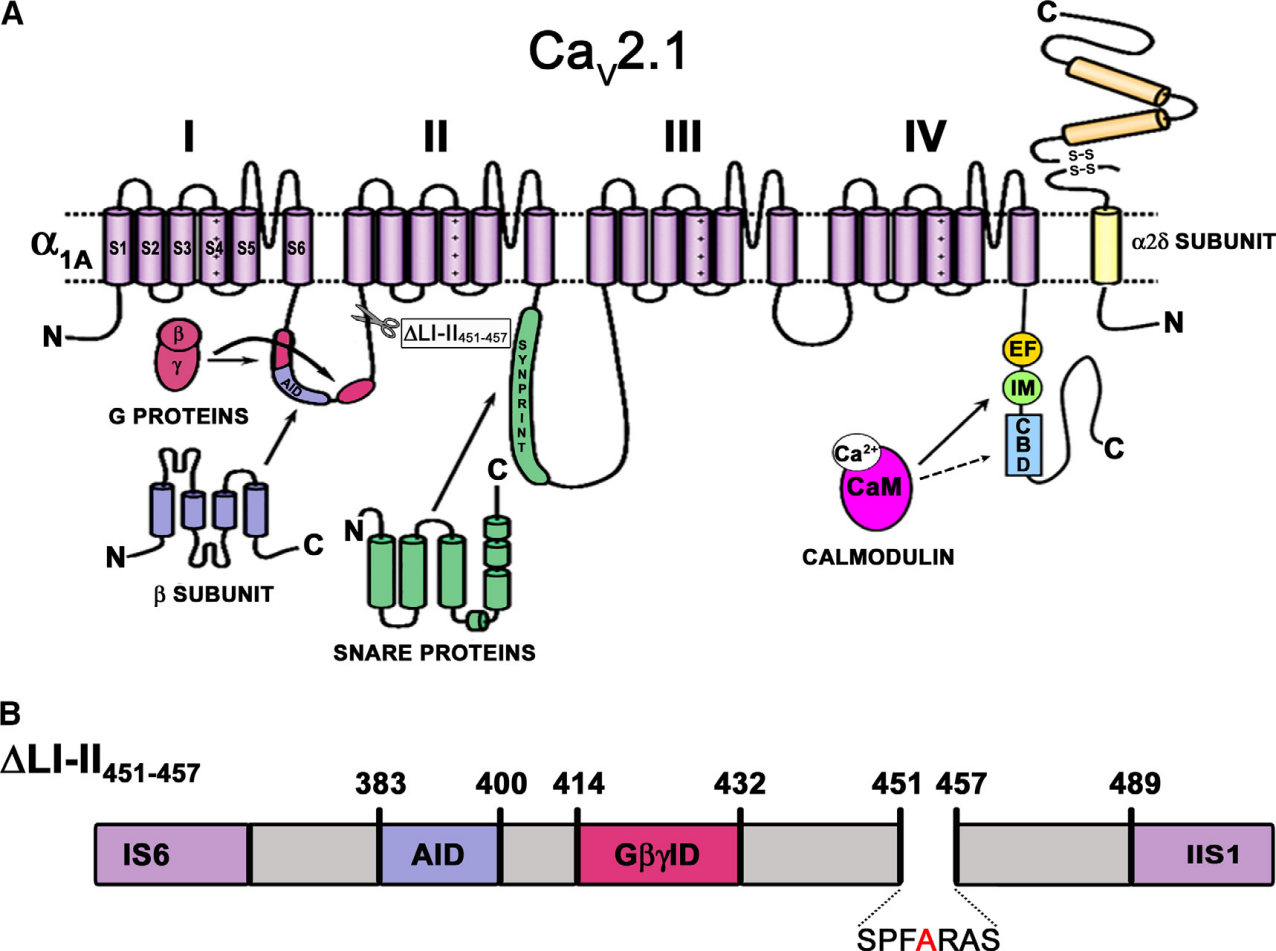
Location of the known *α*_1A_ molecular determinants for the binding of the main intracellular proteins that modulate Ca_V_2.1 channel inactivation. (A) Schematic representation of the secondary structure of the Ca_V_2.1 *α*_1A_ channel subunit, showing the location of the known *α*_1A_ molecular determinants for the binding of three cytosolic proteins involved in the regulation of Ca_V_2.1 channel inactivation: (1) regulatory *β* subunits (which binds to the *α*‐interaction domain or AID, located in the cytoplasmic loop connecting domains I and II (LI‐II) of *α*_1A_); (2) SNARE proteins (that interacts with the *synprint* site, within the intracellular loop connecting domains II and III (LII‐III) of *α*_1A_); and (3) the Ca^2+^‐calmodulin (Ca^2+^‐CaM) complex (which binds to the IQ‐like motif and the Calmodulin‐Binding Domain (CBD) site, at the carboxyl tail of *α*_1A_). (B) Location of the *α*_1A_ LI‐II deletion around the A454 residue (ΔLI‐II_451–457_) (also depicted at panel A).

Ca^2+^ entry through the high‐voltage‐activated (HVA) Ca_V_2.x channels (mainly Ca_V_2.1 [P/Q‐type] channels) into presynaptic nerve terminals supports a transient Ca^2+^ microdomain that is essential for synaptic exocytosis leading to the fast release of classical neurotransmitters (Catterall 2011). To ensure fast and efficient neurotransmitter release, the vesicle‐docking/release machinery must be located near the pathway of Ca^2+^ entry. In many cases, this close localization is achieved by direct interaction of soluble N‐ethylmaleimide‐sensitive factor attachment protein receptor (SNARE) proteins with the Ca^2+^ channel pore‐forming *α*_1_ subunit, which consists of four repeated domains (I‐IV) each containing six transmembrane regions (S1–S6) with a voltage sensor (S1–S4) and a pore region (S5, P‐loop, and S6). Indeed, syntaxin‐1A/1B, SNAP‐25, and synaptotagmin‐1 specifically interact with Ca_V_2.1 and Ca_V_2.2 channels by binding to a synaptic protein interaction site (*synprint*) located within the intracellular loop connecting domains II and III (LII‐III) of the channels (Sheng et al. 1994, 1997; Rettig et al. 1996; Kim and Catterall 1997; Jarvis et al. 2002) (Fig. 1A). Furthermore, it has been suggested that exocytosis is activated even before Ca^2+^ entry, by conformational changes triggered during Ca^2+^ binding at the open Ca_V_ channel pore, which are transmitted from the channel to specific residues of Ca_V_‐interacting SNARE proteins, accounting for the rapid time frame of evoked release (Atlas 2013; Bachnoff et al. 2013). Whichever the case, perhaps an equally important consequence of SNARE protein interaction with the Ca^2+^ channel is the modulation of presynaptic Ca^2+^ channel activity, thus fine‐tuning the amount of Ca^2+^ that binds to the pore, enters the synaptic terminal, and determines synaptic transmission strength. Specifically, the binding of syntaxin‐1A and SNAP‐25 to Ca_V_2.1 and Ca_V_2.2 *α*_1_ subunits shifts the voltage dependence of steady‐state inactivation toward more negative membrane potentials following trains of brief depolarizing pulses to reduce channel availability, without affecting channel activation properties (Bezprozvanny et al. 1995; Zhong et al. 1999). Such inhibition is reverted, and channel activity fully restored, by synaptotagmin (Zhong et al. 1999). Thus, Ca_V_2.x‐SNAREs interaction seems to optimize neurotransmission by favoring Ca^2+^ entry through channels presenting docked synaptic vesicles. Accordingly, the disruption of such functional interaction compromises not just vesicle exocytosis in vitro (Mochida et al. 2003; Harkins et al. 2004), but also synaptic transmission and the SNARE‐mediated inhibitory modulation of Ca_V_2.x channels in vivo (Mochida et al. 1996; Rettig et al. 1997; Zamponi 2003; Keith et al. 2007). Moreover, human *α*_1A_ mutations impairing the functional interaction between Ca_V_2.1 channels and SNARE proteins have clinical relevance in the context of ataxia and the phenotypic expression of both migraine with aura and hemiplegic migraine (Cricchi et al. 2007; Serra et al. 2010; Condliffe et al. 2013).

Beyond the important anchoring function of the *synprint* site in the SNARE‐mediated modulation of Ca_V_2.x channel gating, the involvement of other molecular domains has been proposed. Hence, deletions within the LII‐III intracellular loop of the Ca_V_2.2 *α*_1B_ channel subunit that completely eliminate the *synprint* site reduce but not abolish channel modulation by syntaxin, and syntaxin mutations that have no effect on binding affinity to *α*_1B_‐*synprint* prevent the SNARE‐mediated regulation of Ca_V_2.2 channel inactivation (Bezprozvanny et al. 2000). Besides, the A454T mutation (placed in the intracellular loop connecting domains I and II (LI‐II) of the Ca_V_2.1 *α*_1A_ channel subunit, and associated to both early‐onset progressive ataxia (Cricchi et al. 2007) and the relief of migraine aura symptoms (Serra et al. 2010)) prevents the negative modulation of Ca_V_2.1 channels by SNARE proteins and decreases channel coupling to exocytosis, thus revealing the importance of LI‐II structural integrity in the Ca_V_2.1‐SNAREs functional interaction (Serra et al. 2010).

The molecular mechanism by which LI‐II influences Ca_V_2.1‐SNAREs functional interaction is unknown. However, it is well established that LI‐II plays a determinant role in the regulation of Ca_V_2.1 channel activity (Buraei and Yang 2010). In this sense, it has been suggested that conformational changes induced at LI‐II by the binding of functionally different regulatory *β* subunits not only determine the degree of voltage‐dependent inactivation but also the extent of a Ca^2+^‐dependent inactivation component (mediated by the binding of Ca^2+^‐calmodulin to two adjacent sites in the carboxyl tail [C‐tail] of the *α*_1A_ subunit: the IQ‐like motif and the Calmodulin‐Binding Domain [CBD] site) (Lee et al. 2000; DeMaria et al. 2001; Cens et al. 2006) (Fig. 1A). Interestingly, disruption of Ca_V_2.1 modulation by calmodulin and related Ca^2+^ sensor proteins by mutation of the IQ‐like motif has been reported to impair long‐term potentiation and spatial learning and memory in mice (Nanoua et al. 2016).

Altogether, it draws a complex scenario in which Ca_V_2.1 inactivation is produced by LI‐II and modulated by: *β* channel subunits interacting with LI‐II, SNARE proteins binding to the *synprint* site at the LII‐III but requiring the integrity of LI‐II, and Ca^2+^‐calmodulin attached to the C‐tail of the *α*_1A_ subunit.

To better understand the role of LI‐II in Ca_V_2.1‐SNAREs functional interaction, we analyzed the modulation of Ca_V_2.1 inactivation by syntaxin‐1A under intermediate and high Ca^2+^‐buffering conditions, in the presence of functionally different regulatory *β* subunits (*β*_2a_ or *β*_3_) and distinct human α_1A_ constructs containing either a LI‐II deletion around the A454 residue, mutations in the IQ‐like region, or a CBD deletion. Our results reveal a cross talk between different pathways involved in the modulation of Ca_V_2.1 inactivation, showing that regulation by syntaxin‐1A of the human Ca_V_2.1 channel activity requires both the integrity of α_1A_ LI‐II and the lack of a Ca^2+^‐dependent component in the channel steady‐state inactivation.

## Methods

### cDNA constructs and site‐directed mutagenesis

cDNA of the human voltage‐gated Ca^2+^ (Ca_V_2.1) channel α_1A_ subunit (originally cloned into a pCMV vector) was a gift from Professor J. Striessnig (University of Innsbruck, Austria). cDNAs of the rabbit *α*_2_ *δ* and rat *β*_3_ and *β*_2a_ regulatory subunits, and syntaxin‐1A (subcloned into a pcDNA3 expression vector) were gifts from Dr. L. Birnbaumer (National Institutes of Health, North Carolina, USA) and Dr. J. Blasi (Universitat de Barcelona, Spain). Ca_V_2.1 *α*_1A_ mutant subunits (ΔLI‐II_451–457_, IM/EE_1964;1965_, ΔCBD_2020‐end_) were generated using site‐directed mutagenesis (GenScript Corporation, Piscatway, NJ). All cDNA clones used in this study were sequenced in full to confirm their integrity.

### Heterologous expression and electrophysiology

HEK 293 cells were transfected using a linear polyethylenimine (PEI) derivative, the polycation ExGen500 (Fermentas Inc., Hanover, Maryland, USA) as previously reported (eight equivalents PEI/3.3 *μ*g DNA/dish) (Serra et al. 2010). For transfection, α_1A_ (wild‐type [WT] or mutants), *β*_3_ or *β*_2a_, *α*_2_ *δ*, and EGFP (transfection marker) cDNA constructs were used at a ratio of 1:1:1:0.3. When required, syntaxin‐1A was also cotransfected at the same ratio as Ca_V_2.1 channel subunit cDNAs. Electrophysiological recordings were obtained from EGFP‐positive cells 24–48 h after transfection at room temperature (22–24°C).

Ca^2+^ currents (I_Ca_^2+^) through WT or mutant Ca_V_2.1 channels containing *β*_3_ or *β*_2a_ regulatory subunits were recorded in the whole‐cell configuration of the patch‐clamp technique, using a D‐6100 Darmstadt amplifier (List Medical, Germany). Pipettes had a resistance of 2–3 MΩ when filled with a solution containing (in mmol/L): 140 CsCl, 1 EGTA (intermediate Ca^2+^‐buffering condition) or 10 BAPTA (high Ca^2+^‐buffering condition), 4 Na_2_ATP, 0.1 Na_3_GTP, and 10 Hepes (pH 7.2–7.3 and 290–300 mOsmol/L). The external solution contained (in mmol/L): 140 tetraethylammonium‐Cl (TEACl), 3 CsCl, 2.5 CaCl_2_, 1.2 MgCl_2_, 10 Hepes, and 10 D‐glucose (pH 7.4 and 300–310 mOsmol/L). Previous work demonstrates that high levels of intracellular Ca^2+^ chelators (e.g., 10 mmol/L EGTA or 10 mmol/L BAPTA) impair Ca_V_2.1 inactivation in a similar way as when replacing extracellular Ca^2+^ by Ba^2+^ (Lee et al. 2000). Such observation strongly suggest that chelator effect is due to Ca^2+^ buffering, ruling out any unwanted direct action of the Ca^2+^ chelator itself on channel inactivation. pClamp8 software (Molecular Devices, USA) was used for pulse generation, data acquisition, and subsequent analysis.

Steady‐state inactivation was estimated by measuring peak Ca^2+^ currents in response to a 50 ms (or 10 ms, when using the *α*_1A_ IM/EE mutant subunit) depolarizing test pulse (to +20 mV) from a holding of −80 mV, following 30‐sec steps to various holding potentials (conditioning pulses) between −80 and +20 mV (Fig. 1A). Between the 30‐sec conditioning depolarizations and the test pulse we employed a 20‐msec interpulse to the holding potential, which does not allow detectable recovery from inactivation of Ca_V_2.x channels (Degtiar et al. 2000). This kind of protocol has been reported to detect significant increase in Ca_V_2.x steady‐state inactivation induced by syntaxin‐1A (i.e., a left shift of V_1/2_ _inact_ to more negative voltages by ~6 mV) (Degtiar et al. 2000). On the contrary, when using shorter (few seconds) conditioning pulses, the influence of syntaxin‐1A on Ca_V_2.x channel gating was barely detectable (Degtiar et al. 2000). These results are consistent with the action of SNAREs on slow rather than fast channel inactivation (Degtiar et al. 2000). As described in detail previously (Serra et al. 2010), normalized *I*_Ca_^2+^ persistent currents were fitted to the following Boltzmann equation in order to obtain half‐maximal voltage (*V* _1/2_ _inact_) and slope factor (*k*_inact_) for steady‐state inactivation:

(1) $$\frac{I}{I\text{max}}\;=\;\frac{1}{1+e^{\frac{V-V1/2\;\text{inact}}{\text{kinact}}}}$$

### Statistics

Data are presented as the means ± SEM, and *n* represents the number of cells recorded for each experimental condition. Statistical significance was tested using one‐way Analysis of Variance (ANOVA) followed by a Bonferroni post hoc test. Differences were considered significant if *P* < 0.05. All statistical comparisons were performed using the GraphPad Instat software. All data are sampled from Gaussian (normal) distributions (tested using the method Kolmogorov and Smirnov).

## Results

**Figure 2:**
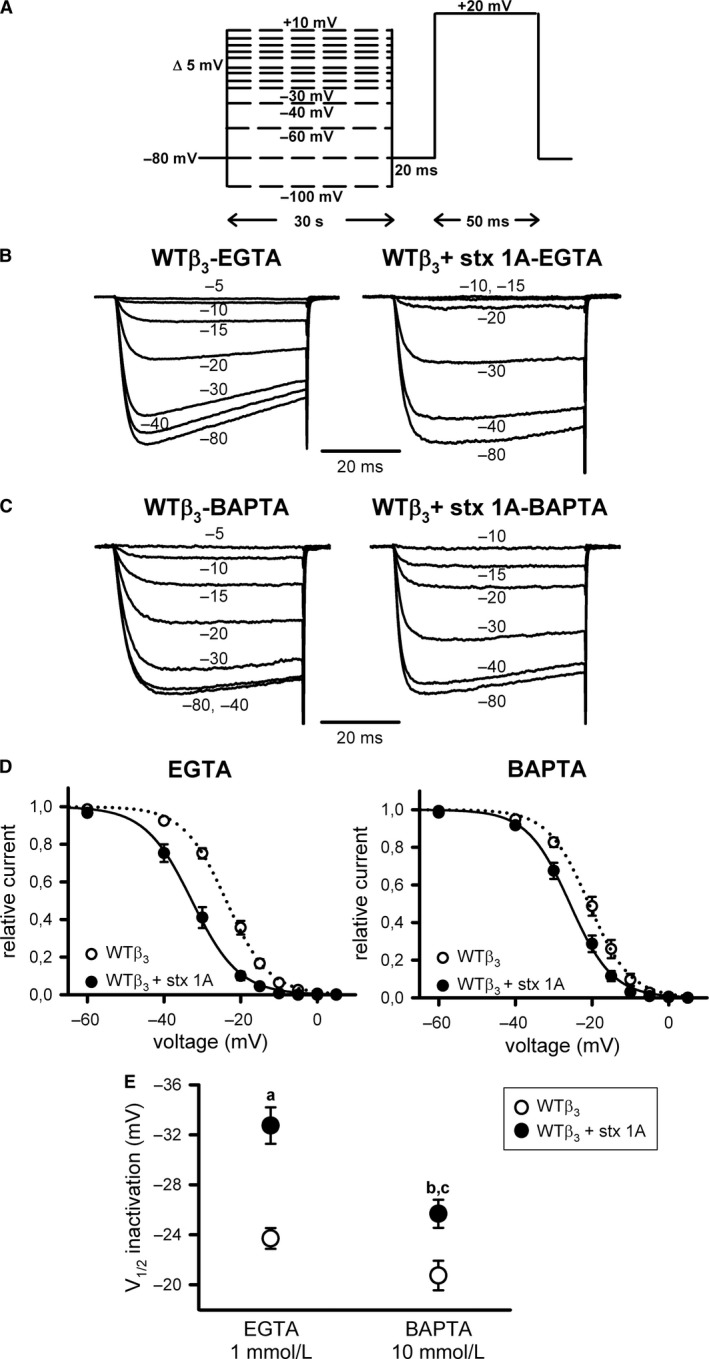
Steady‐state inactivation of Ca_V_2.1 channels containing the regulatory *β*_3_ subunit is independent of intracellular Ca^2+^ signaling and is favored by syntaxin‐1A (A) Voltage protocol for the study of Ca_V_2.1 channels steady‐state inactivation (see Methods for further details). Representative normalized Ca^2+^ current traces recorded at intermediate (1 mmol/L EGTA) (B) or high (10 mmol/L BAPTA) (C) intracellular Ca^2+^‐buffering conditions from a HEK 293 cell expressing Ca_V_2.1 channels composed of wild‐type *α*_1A_, *β*_3_, and *α*_2_ *δ* subunits (WT *β*_3_) either in the absence (left) or presence (right) of syntaxin‐1A (stx 1A). Currents were elicited by 50 ms depolarizing steps to +20 mV applied after 30‐sec depolarizing prepulses to the indicated voltages. Amplitudes of currents elicited by test pulses to +20 mV after the different prepulses were normalized to the maximum current amplitude obtained after a 30‐sec prepulse to −80 mV in order to generate the corresponding mean steady‐state inactivation curves (D), which were fitted to a single Boltzmann function (see Methods, eq. (1)) to estimate the half‐inactivation potentials (V_1/2_ inactivation) (E) for WT Ca_V_2.1 channels containing the *β*_3_ subunit (WT *β*_3_) in the absence (open circles) or presence (filled circles) of syntaxin‐1A (stx 1A), at the above indicated intracellular Ca^2+^‐buffering conditions. Average V_1/2_ _inact_ and k_inact_ values at intermediate Ca^2+^‐buffering condition (1 mmol/L EGTA) were (in mV): WT *β*_3_ (open circles, *n* = 18) −23.7 ± 0.83 and −5.16 ± 0.24; WT *β*_3_ + stx 1A (filled circles, *n* = 12) −32.74 ± 1.46 and −5.5 ± 0.19, respectively. At high Ca^2+^‐buffering condition (10 mmol/L BAPTA), average V_1/2_ _inact_ and k_inact_ values were (in mV): WT *β*_3_ (open circles, *n* = 12) −20.74 ± 1.18 and −4.78 ± 0.16; WT *β*_3_ + stx 1A (filled circles, *n* = 8) −25.68 ± 1.13 and −5.25 ± 0.21, respectively. a and b: *P* < 0.001 and *P* < 0.05 versus the corresponding control condition (absence of syntaxin‐1A), respectively; c: *P* < 0.01 when compared to the intermediate Ca^2+^‐buffering condition (1 mmol/L EGTA). No significant difference was found for k_inact_ values (ANOVA *P* = 0.17).

The impact of the SNARE protein syntaxin‐1A on the steady‐state inactivation of Ca^2+^ currents (I_Ca_^2+^) through wild‐type (WT) Ca_V_2.1 channel containing the regulatory *β*_3_ subunit (WT*β*_3_), was measured at intermediate (1 mmol/L EGTA) and high (10 mmol/L BAPTA) intracellular Ca^2+^‐buffering conditions to evaluate its calcium dependency. In both conditions, syntaxin‐1A expression favored channel steady‐state inactivation, as indicated by a significant left shift of V_1/2_ _inact_ to more negative voltages (by ~5–9 mV) (Fig. 2B–E; Table 1). It must be noted that steady‐state inactivation of WT*β*_3_ channels was poorly dependent on intracellular Ca^2+^ concentration, as not significant differences were found when comparing I_Ca_^2+^ V_1/2_ _inact_ values obtained at intermediate and high intracellular Ca^2+^‐buffering conditions in the absence of syntaxin‐1A (Fig. 2B and C (left panels), D and E (open circles); Table 1).

**Table 1:**
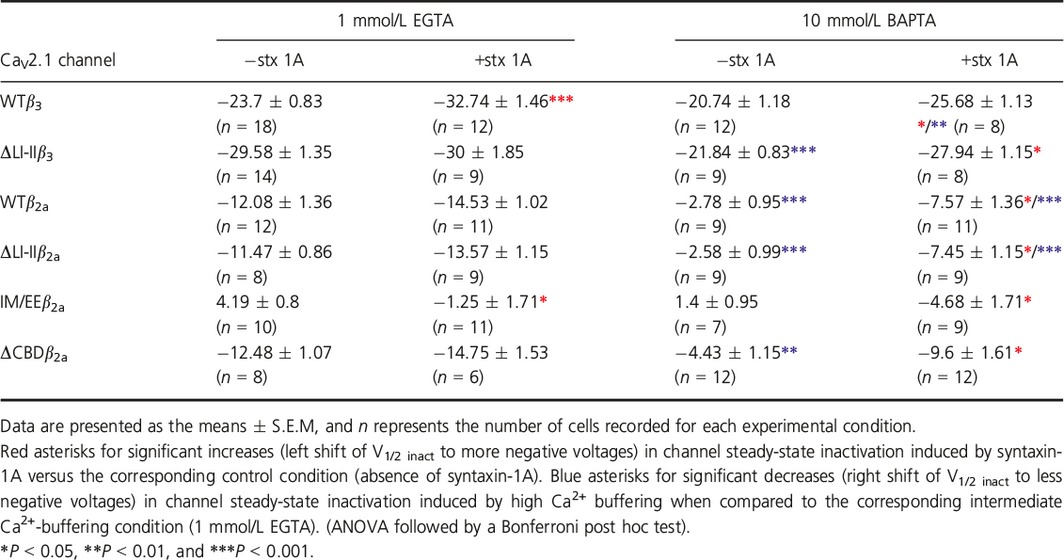
Summary of half‐maximal voltage for steady‐state inactivation (V1/2 inact) values of the different CaV2.1 channels analyzed under intermediate (1 m EGTA) and high (10 mmol/L BAPTA) Ca^2+^ ‐buffering conditions in the absence (−stx 1A) or presence (+stx 1A) of syntaxin‐1A

**Figure 3:**
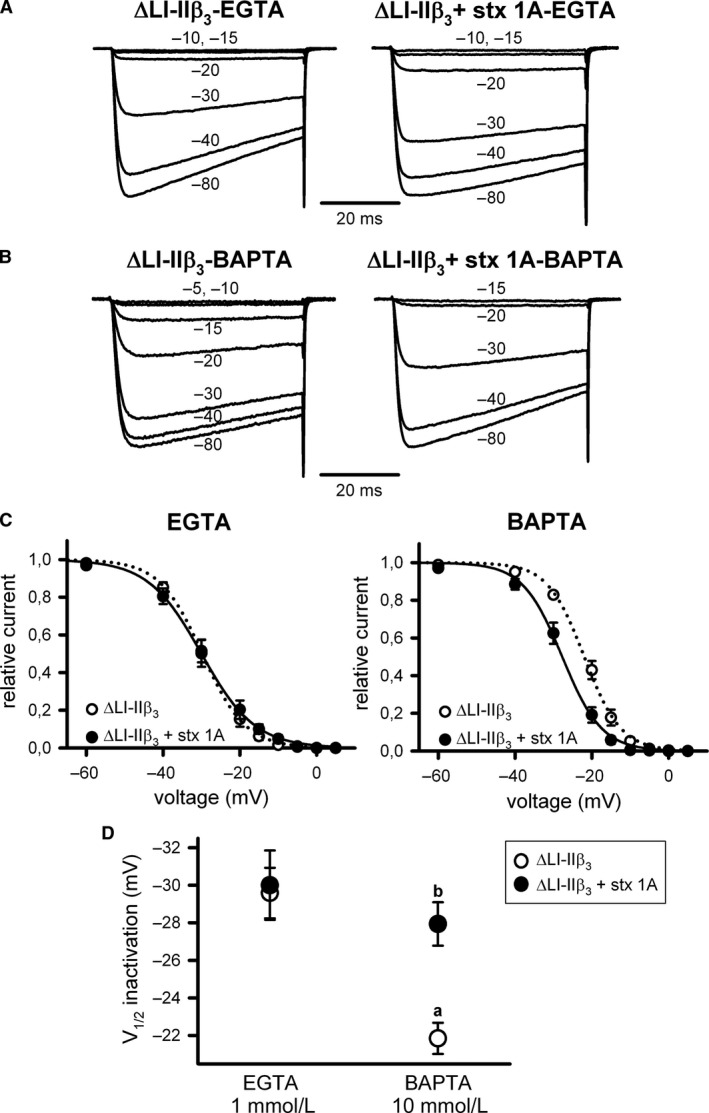
Δ451‐457 at *α*_1A_ LI‐II promotes a Ca^2+^‐dependent component in the steady‐state inactivation of Ca_V_2.1 channels containing the auxiliary *β*_3_ subunit, and it prevents syntaxin‐1A‐mediated modulation. Typical normalized Ca^2+^ current traces recorded at intermediate (1 mmol/L EGTA) (A) or high (10 mmol/L BAPTA) (B) intracellular Ca^2+^‐buffering conditions from a HEK 293 cell expressing Ca_V_2.1 channels composed of mutant ΔLI‐II_451–457_ *α*_1A_, *β*_3_, and *α*_2_ *δ* subunits (ΔLI‐II *β*_3_) either in the absence (left) or presence (right) of syntaxin‐1A (stx 1A). Currents were elicited by 50‐ms depolarizing steps to +20 mV applied after 30‐sec depolarizing prepulses to the shown voltages. Corresponding mean normalized steady‐state inactivation curves (C), and derived V_1/2_ inactivation (D) for ΔLI‐II_451–457_ Ca_V_2.1 mutant channels containing the *β*_3_ subunit (ΔLI‐II *β*_3_) in the absence (open circles) or presence (filled circles) of syntaxin‐1A (stx 1A), at the above indicated intracellular Ca^2+^‐buffering conditions. Average V_1/2_ _inact_ and k_inact_ values at intermediate Ca^2+^‐buffering condition (1 mmol/L EGTA) were (in mV): ΔLI‐II *β*_3_ (open circles, *n* = 14) −29.58 ± 1.35 and −4.73 ± 0.16; ΔLI‐II *β*_3_ + stx 1A (filled circles, *n* = 9) −30 ± 1.85 and −5.73 ± 0.35, respectively. At high Ca^2+^‐buffering condition (10 mmol/L BAPTA), average V_1/2_ _inact_ and k_inact_ values were (in mV): ΔLI‐II *β*_3_ (open circles, *n* = 9) −21.84 ± 0.83 and −4.94 ± 0.47; ΔLI‐II *β*_3_ + stx 1A (filled circles, *n* = 8) −27.94 ± 1.15 and −4.97 ± 0.33, respectively. a: *P* < 0.001 when compared to the intermediate Ca^2+^‐buffering condition (1 mmol/L EGTA); b: *P* < 0.05 versus the corresponding control condition (absence of syntaxin‐1A). No significant difference was found for k_inact_ values (ANOVA *P* = 0.14).

Interestingly, the introduction of a small deletion around the A454 residue at the first intracellular loop of the pore‐forming α_1A_ subunit (ΔLI‐II_451–457_) (Fig. 1) made the steady‐state inactivation of *β*_3_‐containing Ca_V_2.1channels (ΔLI‐II*β*_3_) Ca^2+^‐dependent. On one hand, I_Ca_^2+^ inactivation was reduced (with a significant ~8 mV right shift in the V_1/2_ _inact_) by increasing the buffering of intracellular Ca^2+^ (Fig. 3A and B (left panels), C and D (open circles); Table 1). On the other hand, such α_1A_ LI‐II deletion removed the modulatory action of syntaxin‐1A on the steady‐state inactivation of Ca_V_2.1 channels containing *β*_3_ at intermediate Ca^2+^‐buffering condition (Fig. 3A, C [left panel], and D; Table 1), when Ca^2+^ entry through the channel promotes inactivation. The effect of syntaxin‐1A on the inactivation of the ΔLI‐II*β*_3_ channel was recovered (V_1/2_ _inact_ was significantly left‐shifted by ~6 mV) by increasing intracellular Ca^2+^ buffering to abrogate the novel LI‐II deletion‐induced Ca^2+^‐dependent component of inactivation (Fig. 3B and C [right panel], D; Table 1).

**Figure 4:**
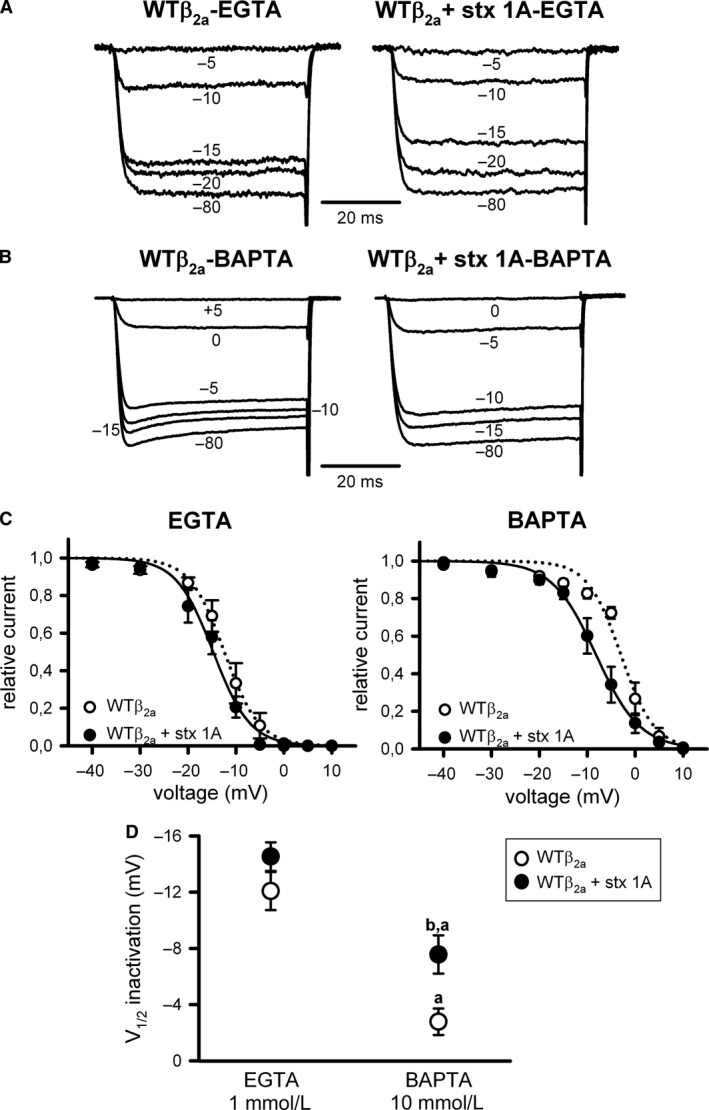
Steady‐state inactivation of Ca_V_2.1 channels containing the regulatory *β*_2a_ subunit presents a Ca^2+^‐dependent component and no regulation by syntaxin‐1A. Illustrative normalized Ca^2+^ current traces recorded at intermediate (1 mmol/L EGTA) (A) or high (10 mmol/L BAPTA) (B) intracellular Ca^2+^‐buffering conditions from a HEK 293 cell expressing Ca_V_2.1 channels composed of WT *α*_1A_, *β*_2a_, and *α*_2_ *δ* subunits (WT *β*_2a_) either in the absence (left) or presence (right) of syntaxin‐1A (stx 1A). Currents were elicited by 50‐ms depolarizing steps to +20 mV applied after 30‐sec depolarizing prepulses to the indicated voltages. Corresponding mean normalized steady‐state inactivation curves (C), and estimated V_1/2_ inactivation (D) for ΔLI‐II_451–457_ Ca_V_2.1 mutant channels containing the *β*_2a_ subunit (WT *β*_2a_) in the absence (open circles) or presence (filled circles) of syntaxin‐1A (stx 1A), at the above indicated intracellular Ca^2+^‐buffering conditions. Average V_1/2_ _inact_ and k_inact_ values at intermediate Ca^2+^‐buffering condition (1 mmol/L EGTA) were (in mV): WT *β*_2a_ (open circles, *n* = 12) −12.08 ± 1.36 and −2.43 ± 0.41; WT *β*_2a_ + stx 1A (filled circles, *n* = 11) −14.53 ± 1.02 and −2.33 ± 0.28, respectively. At high Ca^2+^‐buffering condition (10 mmol/L BAPTA), average V_1/2_ _inact_ and k_inact_ values were (in mV): WT *β*_2a_ (open circles, *n* = 9) −2.78 ± 0.95 and −3.1 ± 0.52; WT *β*_2a_ + stx 1A (filled circles, *n* = 11) −7.57 ± 1.36 and −3.15 ± 0.55, respectively. a: *P* < 0.001 when compared to the intermediate Ca^2+^‐buffering condition (1 mmol/L EGTA); b: *P* < 0.05 versus the corresponding control condition (absence of syntaxin‐1A). No significant difference was found for k_inact_ values (ANOVA *P* = 0.44).

**Figure 5:**
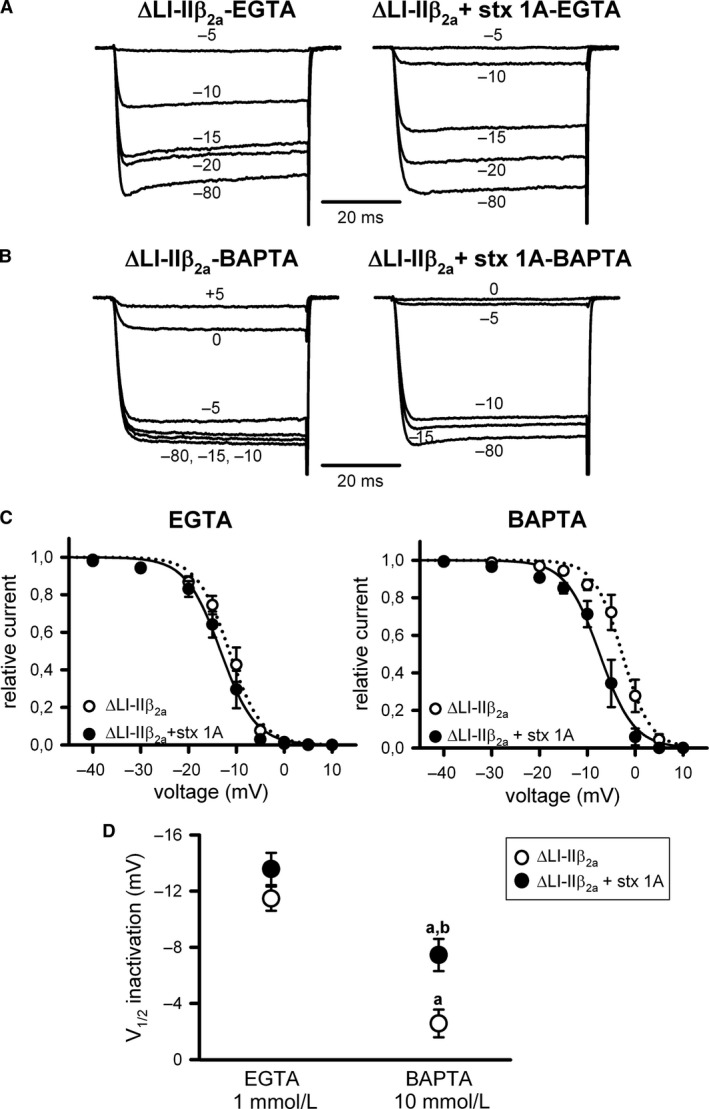
Steady‐state inactivation of Ca_V_2.1 channels formed by mutant *α*_1A_ ΔLI‐II_451–457_ and *β*_2a_ subunits remains Ca^2+^‐dependent and syntaxin‐1A‐insensitive. Representative normalized Ca^2+^ current traces recorded at intermediate (1 mmol/L EGTA) (A) or high (10 mmol/L BAPTA) (B) intracellular Ca^2+^‐buffering conditions from a HEK 293 cell expressing Ca_V_2.1 channels composed of mutant ΔLI‐II_451–457_ *α*_1A_, *β*_2a_, and *α*_2_ *δ* subunits (ΔLI‐II *β*_2a_) either in the absence (left) or presence (right) of syntaxin‐1A (stx 1A). Currents were elicited by 50‐ms depolarizing steps to +20 mV applied after 30‐sec depolarizing prepulses to the indicated voltages. Amplitudes of currents elicited by test pulses to +20 mV after the different prepulses were normalized to the maximum current amplitude obtained after a 30‐sec prepulse to −80 mV in order to generate the corresponding mean steady‐state inactivation curves (C), which were fitted to a single Boltzmann function (see Methods, eq. (1)) to estimate the half‐inactivation potentials (V_1/2_ inactivation) (D) for mutant ΔLI‐II_451–457_ Ca_V_2.1 mutant channels containing the *β*_2a_ subunit (ΔLI‐II *β*_2a_) in the absence (open circles) or presence (filled circles) of syntaxin‐1A (stx 1A), at the above indicated intracellular Ca^2+^‐buffering conditions. Average V_1/2_ _inact_ and k_inact_ values at intermediate Ca^2+^‐buffering condition (1 mmol/L EGTA) were (in mV): ΔLI‐II *β*_2a_ (open circles, *n* = 8) −11.47 ± 0.86 and −2.87 ± 0.5; ΔLI‐II *β*_2a_ + stx 1A (filled circles, *n* = 9) −13.57 ± 1.15 and −2.73 ± 0.44, respectively. At high Ca^2+^‐buffering condition (10 mmol/L BAPTA), average V_1/2_ _inact_ and k_inact_ values were (in mV): ΔLI‐II *β*_2a_ (open circles, *n* = 9) −2.58 ± 0.99 and −2.05 ± 0.43; ΔLI‐II *β*_2a_ + stx 1A (filled circles, *n* = 9) −7.45 ± 1.15 and −2.12 ± 0.51, respectively. a: *P* < 0.001 when compared to the intermediate Ca^2+^‐buffering condition (1 mmol/L EGTA); b: *P* < 0.01 versus the corresponding control condition (absence of syntaxin‐1A). No significant difference was found for k_inact_ values (ANOVA *P* = 0.51).

**Figure 6:**
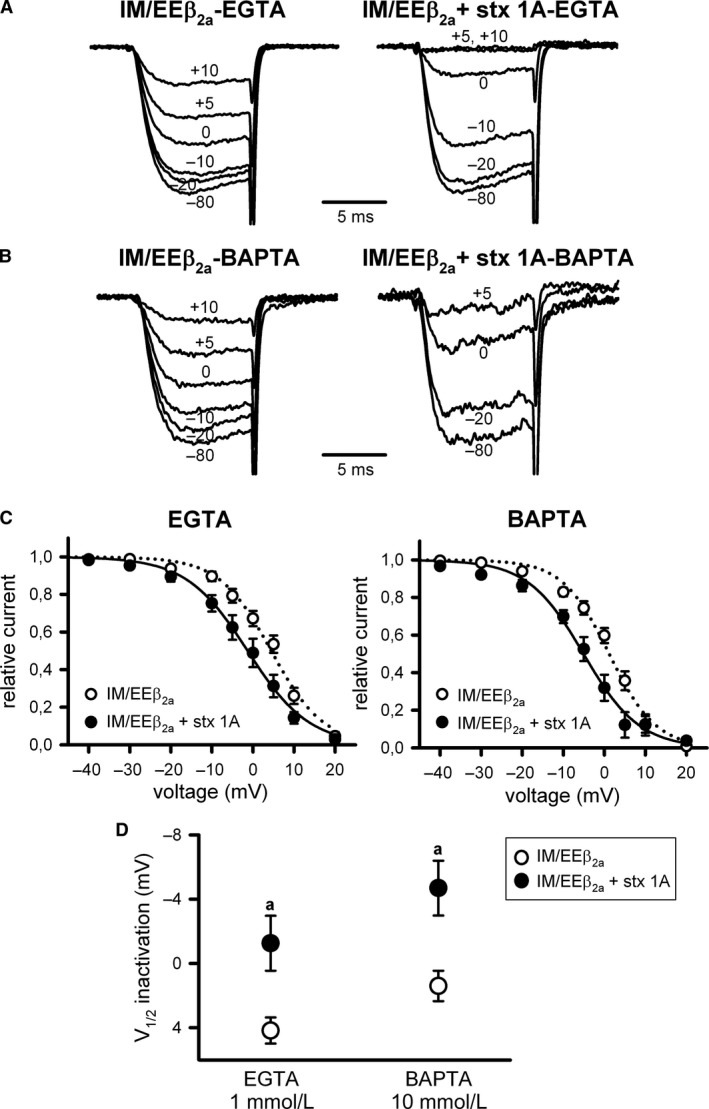
*α*_1A_ IQ‐like motif mutation (IM/EE) remove the Ca^2+^‐dependent component in the steady‐state inactivation of *β*_2a_‐containing Ca_V_2.1 channels, and it allows modulation by syntaxin‐1A. Representative normalized Ca^2+^ current traces recorded at intermediate (1 mmol/L EGTA) (A) or high (10 mmol/L BAPTA) (B) intracellular Ca^2+^‐buffering conditions from a HEK 293 cell expressing Ca_V_2.1 channels composed of mutant IM/EE *α*_1A_, *β*_2a_, and *α*_2_ *δ* subunits (IM/EE *β*_2a_) either in the absence (left) or presence (right) of syntaxin‐1A (stx 1A). Currents were elicited by 10‐ms depolarizing steps to +20 mV applied after 30‐sec depolarizing prepulses to the shown voltages. Corresponding mean normalized steady‐state inactivation curves (C), and derived V_1/2_ inactivation (D) for IM/EE Ca_V_2.1 mutant channels containing the *β*_2a_ subunit (IM/EE *β*_2a_) in the absence (open circles) or presence (filled circles) of syntaxin‐1A (stx 1A), at the above indicated intracellular Ca^2+^‐buffering conditions. Average V_1/2_ _inact_ and k_inact_ values at intermediate Ca^2+^‐buffering condition (1 mmol/L EGTA) were (in mV): IM/EE *β*_2a_ (open circles, *n* = 10) 4.19 ± 0.8 and ‐6.17 ± 0.5; IM/EE *β*_2a_ + stx 1A (filled circles, *n* = 11) −1.25 ± 1.71 and −6.44 ± 0.34, respectively. At high Ca^2+^‐buffering condition (10 mmol/L BAPTA), average V_1/2_ _inact_ and k_inact_ values were (in mV): IM/EE *β*_2a_ (open circles, *n* = 7) 1.4 ± 0.95 and ‐5.8 ± 0.62; IM/EE *β*_2a_ + stx 1A (filled circles, *n* = 9) −4.68 ± 1.71 and ‐6.21 ± 0.42, respectively. a: *P* < 0.05 versus the corresponding control condition (absence of syntaxin‐1A). No significant difference was found for k_inact_ values (ANOVA *P* = 0.82).

As widely reported before (for a review see Buraei and Yang 2010), Ca_V_2.1 inactivation was substantially right‐shifted to more depolarized potentials for *β*_2a_‐containing than for *β*_3_‐containing channels (Fig. 4 vs. Fig. 2; Table 1). Under this condition, unlike WT*β*_3_ channels, the steady‐state inactivation of I_Ca_^2+^ through the *β*_2a_‐containing WT Ca_V_2.1 channel (WT*β*_2a_) presented a Ca^2+^‐dependent component (Fig. 4) that is not affected by the deletion in the first intracellular loop of the α_1A_ subunit (ΔLI‐II_451–457_) (Fig. 5). Thus, V_1/2_ _inact_ was significantly shifted to less negative values for both WT*β*_2a_ and ΔLI‐II*β*_2a_ channels (by ~9 mV) when increasing intracellular Ca^2+^ buffering (Fig. 4A and B (left panels), C and D (open circles); Fig. 5A and B (left panels), C and D (open circles); Table 1). Accordingly, such right shift in the voltage dependence of inactivation disappeared once the *β*_2a_‐containing Ca_V_2.1 channel was rendered insensitive to Ca^2+^ by the introduction of a double mutation (IM to EE) at the calmodulin‐binding IQ‐like motif (Fig. 6A and B [left panels], C and D [open circles]; Table 1). Modulation by syntaxin‐1A of WT*β*_2a_ and ΔLI‐II*β*_2a_ I_Ca_^2+^ steady‐state inactivation was occluded when the Ca^2+^‐dependent component was present (Fig. 4A and C [left panel], D; Fig. 5A and C [left panel], D; Table 1). Syntaxin‐1A‐induced left shift of V_1/2_ _inact_ (by ~5–6 mV) was only present when the Ca^2+^‐dependent component of the steady‐state inactivation of *β*_2a_‐containing Ca_V_2.1 channels was removed, either by increasing intracellular Ca^2+^ buffering (Fig. 4B and C [right panel], D; Fig. 5B and C [right panel], D; Table 1) or by introducing the IM/EE double mutation at the IQ‐like motif (Fig. 6A–D; Table 1).

**Figure 7:**
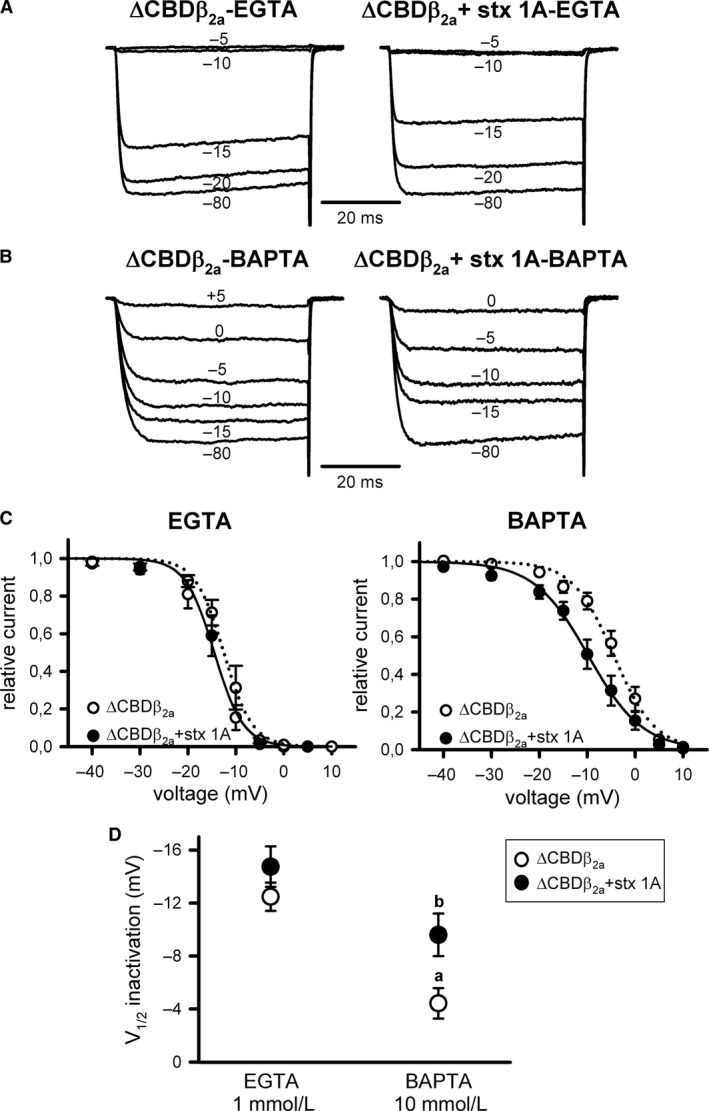
Steady‐state inactivation of Ca_V_2.1 channels formed by mutant *α*_1A_ ΔCBD and *β*_2a_ subunits still shows a Ca^2+^‐dependent component and no regulation by syntaxin‐1A. Typical normalized Ca^2+^ current traces recorded at intermediate (1 mmol/L EGTA) (A) or high (10 mmol/L BAPTA) (B) intracellular Ca^2+^‐buffering conditions from a HEK 293 cell expressing Ca_V_2.1 channels composed of mutant ΔCBD *α*_1A_, *β*_2a_, and *α*_2_ *δ* subunits (ΔCBD *β*_2a_) either in the absence (left) or presence (right) of syntaxin‐1A (stx 1A). Currents were elicited by 50‐ms depolarizing steps to +20 mV applied after 30‐sec depolarizing prepulses to the shown voltages. Corresponding mean normalized steady‐state inactivation curves (C), and derived V_1/2_ inactivation (D) for ΔCBD Ca_V_2.1 mutant channels containing the *β*_2a_ subunit (ΔCBD *β*_2a_) in the absence (open circles) or presence (filled circles) of syntaxin‐1A (stx 1A), at the above indicated intracellular Ca^2+^‐buffering conditions. Average V_1/2_ _inact_ and k_inact_ values at intermediate Ca^2+^‐buffering condition (1 mmol/L EGTA) were (in mV): ΔCBD *β*_2a_ (open circles, *n* = 8) −12.48 ± 1.07 and −2.23 ± 0.25; ΔCBD *β*_2a_ + stx 1A (filled circles, *n* = 6) −14.75 ± 1.53 and −2.59 ± 0.31, respectively. At high Ca^2+^‐buffering condition (10 mmol/L BAPTA), average V_1/2_ _inact_ and k_inact_ values were (in mV): ΔCBD *β*_2a_ (open circles, *n* = 12) −4.43 ± 1.15 and −3.68 ± 0.25; ΔCBD *β*_2a_ + stx 1A (filled circles, *n* = 12) −9.6 ± 1.61 and −4.52 ± 0.5, respectively. a: *P* < 0.01 when compared to the intermediate Ca^2+^‐buffering condition (1 mmol/L EGTA); b: *P* < 0.05 versus the corresponding control condition (absence of syntaxin‐1A). k_inact_ values were significantly higher (*P* < 0.05) at high Ca^2+^‐buffering condition (10 mmol/L BAPTA) than at intermediate Ca^2+^‐buffering condition (1 mmol/L EGTA). The presence of syntaxin‐1A had no significant effect on k_inact_.

Truncation of the α_1A_ carboxyl tail, downstream the IQ‐like motif, to fully remove the distal calmodulin‐binding domain (CBD) site of the *β*_2a_‐containing Ca_V_2.1 channel (ΔCBD*β*_2a_) did not eliminate the Ca^2+^‐dependent component of I_Ca_^2+^ steady‐state inactivation, and V_1/2_ _inact_ was still significantly shifted to less negative values (by ~9 mV) when increasing intracellular Ca^2+^ buffering (Fig. 7A and B (left panels), C and D (open circles); Table 1). At intermediate intracellular Ca^2+^ buffering, the presence of the Ca^2+^‐dependent component in the steady‐state inactivation of ΔCBD*β*_2a_ channels hindered their modulation by syntaxin‐1A (Fig. 7A and C (left panel), D; Table 1), and the SNARE protein only shifted V_1/2_ _inact_ to more negative potentials (by ~5 mV) under high intracellular Ca^2+^ buffering (Fig. 7B and C (right panel), D; Table 1).

## Discussion

Taken together, our results bring to light a functional cross talk between three different signaling pathways regulating Ca_V_2.1 channel steady‐state inactivation: (1) regulatory *β* subunits through their interaction with the α interaction domain (AID) located at the first intracellular loop (LI‐II) of the Ca_V_2.1 pore‐forming *α*_1A_ subunit (Buraei and Yang 2010), (2) Ca^2+^‐calmodulin binding to the IQ‐like motif at the *α*_1A_ C‐tail (DeMaria et al. 2001; Cens et al. 2006; this report), and (3) syntaxin‐1A, quite possibly, via its binding to the *synprint* site at the intracellular loop between domains II and III (LII‐III) of *α*_1A_ (Sheng et al. 1994, 1997; Rettig et al. 1996; Kim and Catterall 1997; Jarvis et al. 2002).

**Figure 8:**
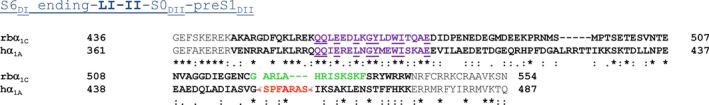
Sequence alignment of intracellular loop between domains I and II (LI‐II) of human Ca_V_2.1 channel *α*_1A_ subunit and rabbit Ca_V_1.2 channel *α*_1C_ subunit. Alignments were performed with Clustal Omega (www.ebi.ac.uk/Tools/msa/clustalo/). AID sequences are shown in purple, and rabbit (rb) *α*_1C_ LI‐II site for Ca^2+^‐calmodulin binding is highlighted in green. Position of the human (h) *α*_1A_ LI‐II deletion around A454 (in red) (ΔLI‐II_451–457_) is shown in orange. “*” identical residues; “:” conservative substitutions (same amino acid group); “.” semi‐conservative substitution (similar shapes). LI‐II residues appear in bold.

As previously reported for fast inactivation (Lee et al. 2000), we observed a substantial Ca^2+^‐dependent component in the steady‐state inactivation of Ca_V_2.1 only in the presence of the palmitoylated, membrane‐anchored *β*_2a_ subunit (which, contrary to other regulatory *β* subunits (such as *β*_1_ or *β*_3_), reduces voltage‐dependent inactivation (Birnbaumer et al. 1998)). In agreement with findings from DeMaria et al. (2001) on Ca_V_2.1 fast inactivation, the Ca^2+^‐dependent component of the steady‐state slow inactivation required the Ca^2+^‐calmodulin‐binding IQ‐like motif, with no detectable role of the previously involved CBD site (Lee et al. 2000). Hence, Ca_V_2.1 Ca^2+^‐dependent steady‐state inactivation was abolished by the introduction of the double mutation IM/EE at the IQ‐like motif, but unaffected by a truncation of the α_1A_ C‐tail, downstream the IQ‐like motif, that fully removes the CBD site. Besides, the Ca^2+^‐dependent component of Ca_V_2.1 steady‐state inactivation seems to depend also on specific conformational changes induced by the binding of the functionally different *β* subunits at the *α*_1A_ LI‐II. Thus, the introduction of a LI‐II deletion (ΔLI‐II_451–457_) downstream the AID, around the A454 residue (of relevance for the modulation of Ca_V_2.1 inactivation by *β* subunits and SNAREs (Serra et al. 2010)), made Ca^2+^‐sensitive the steady‐state inactivation of *β*_3_‐containing Ca_V_2.1 channels. Such Ca_V_2.1 LI‐II deletion affects a fragment of a poorly conserved LI‐II region of 13 amino acids that in the cardiac Ca_V_1.2 channel can bind Ca^2+^‐calmodulin (Pitt et al. 2001) (Fig. 8). Still, removal of this Ca_V_1.2 region (ΔLI‐II_520–532_) did not abolish the Ca^2+^‐dependent component of cardiac channel inactivation (Pitt et al. 2001). This result agrees with our observation that ΔLI‐II_451–457_ had no effect on the Ca^2+^‐dependent inactivation of Ca_V_2.1 channels containing the *β*_2a_ subunit.

Interestingly, syntaxin‐1A was only able to modulate Ca_V_2.1 steady‐state inactivation when the Ca^2+^‐dependent component was absent either because of the presence of *β*_3_ in a channel formed by a *α*_1A_ subunit with unaltered LI‐II, or due to the removal of channel Ca^2+^‐sensitivity by high intracellular Ca^2+^ buffering or by mutation of the IQ‐like motif.

**Figure 9:**
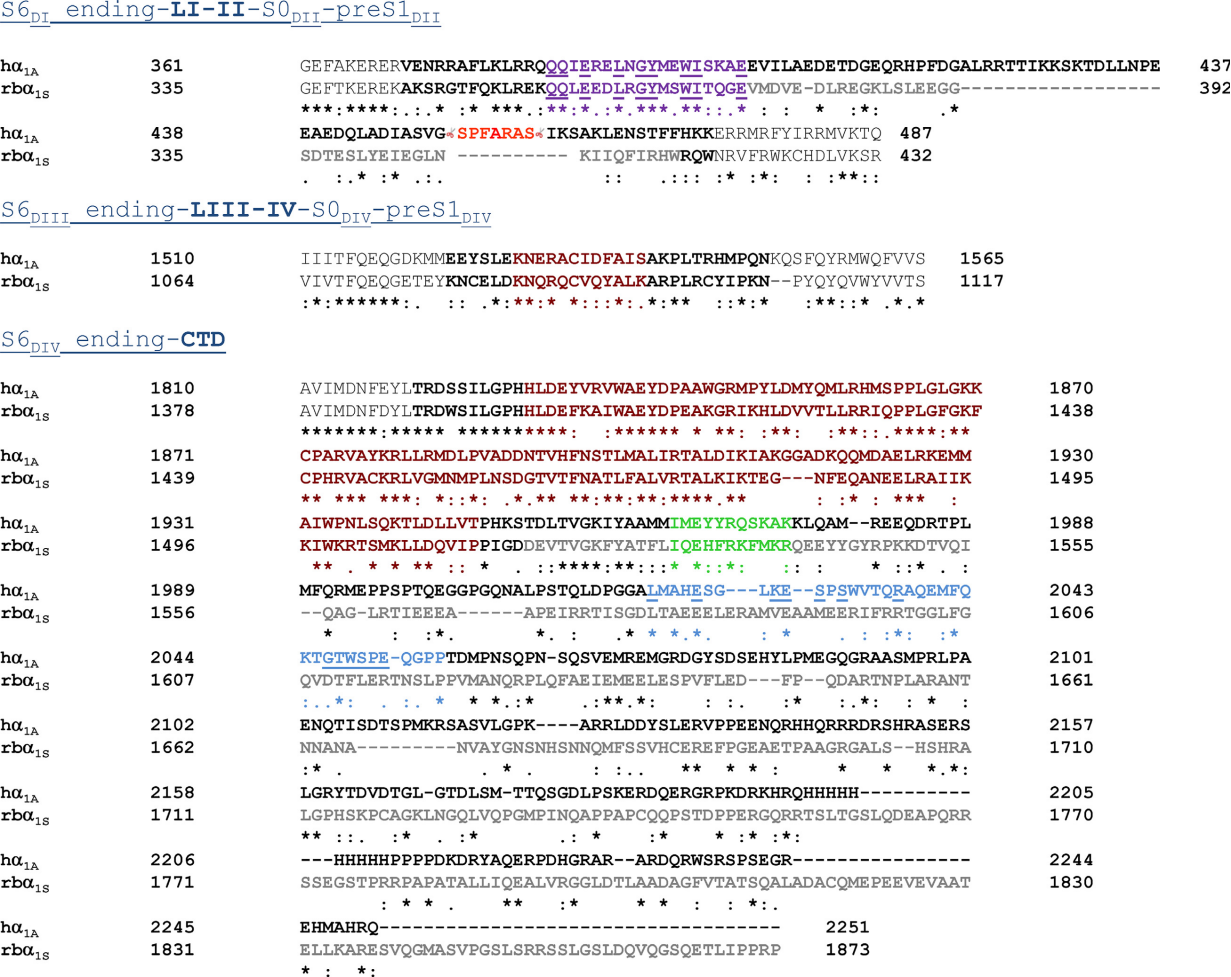
Sequence alignment of intracellular domains (LI‐II, LIII‐IV, and C‐tail) of human Ca_V_2.1 channel *α*_1A_ subunit and rabbit Ca_V_1.1 channel *α*_1S_ subunit. Alignments were performed with Clustal Omega (www.ebi.ac.uk/Tools/msa/clustalo/). AID sequences at LI‐II are shown in purple, and the human (h) *α*_1A_ LI‐II deletion around A454 (in red) (ΔLI‐II_451–457_) is highlighted in orange. Amino acids involved in the physical interaction between LIII‐IV and C‐terminal domain (CTD) of rabbit (rb) *α*_1S_, according to cryo‐EM structural data (Wu et al. 2016), and the homologous sequences in h*α*_1A_ are shown in brown. Sequences of the IQ (rb*α*_1S_) and IQ‐like (h*α*_1A_) motifs are shown in green. h*α*_1A_ CBD sequence is depicted in blue. Cytoplasmic segments that were invisible in the cryo‐EM structure of Ca_V_1.1 rb*α*_1S_ subunit (Wu et al. 2016) are shown in gray. “*” identical residues; “:” conservative substitutions (same amino acid group); “.” semi‐ conservative substitution (similar shapes). LI‐II, LIII‐IV, and CTD residues appear in bold.

Whether the above described functional cross talk is due to a three‐dimensional rearrangement of the involved *α*_1A_ intracellular domains (i.e., LI‐II, LII‐III and C‐tail), and the subsequent alteration of the interaction pattern between them and/or with their interacting partners (regulatory *β* subunits, SNARE proteins, and the Ca^2+^‐calmodulin complex), remains to be elucidated. However, there is evidence that make this hypothesis plausible since it has been reported that N‐tail, intracellular loop between domains III and IV (LIII‐IV) and C‐tail regions of Ca_V_2.x or Ca_V_1.2 *α*_1_ subunits modulate channel inactivation through direct and dynamic interactions with LI‐II, or indirectly via regulatory *β* subunits (Geib et al. 2002; Kim et al. 2004; Stotz et al. 2004). To date, there are no structural data regarding the whole Ca_V_2.1 channel complex that allow us to confirm these physical interactions between *α*_1A_ cytoplasmic domains. Nevertheless, the cryo‐electron microscopy (cryo‐EM) structure of the rabbit Ca_V_1.1 complex, containing the pore‐forming *α*_1S_ and the regulatory *α*_2_ *δ*_1_, *β*_1a_, and *γ* subunits, has been recently resolved with high, near‐atomic (3.6 Å) resolution (Wu et al. 2016). The structural analysis provides an atomic model for a potentially inactivated state of the Ca_V_1.1 channel. In relation to the three‐dimensional arrangement of *α*_1S_ intracellular domains, the structural data locate the AID motif at LI‐II packed in between the regulatory *β*_1a_ subunit and the voltage sensor of *α*_1S_ domain II, and shows the formation of a globular helical domain due to the interaction between LIII‐IV and the proximal C‐tail (upstream the IQ motif) (Wu et al. 2016). The substantial homology between rabbit *α*_1S_ and human *α*_1A_ subunits with regard to residues involved in such LIII‐IV/C‐tail physical interaction (Fig. 9) suggests a similar scenario for the Ca_V_2.1 channel. Unfortunately, several cytoplasmic segments were not visible in the cryo‐EM structure of Ca_V_1.1 *α*_1S_ subunit, and the structure of LI‐II upstream the AID, the whole LII‐III and the C‐tail after residue D1515 (including the IQ motif) could not be resolved (Wu et al. 2016). Therefore, there are no structural data available neither on the possible interaction of LI‐II with either LIII‐IV or the C‐tail, or on any interaction involving LII‐III.

Biochemical experiments with recombinant proteins in vitro strongly indicate that the *synprint* site, located at LII‐III of *α*_1A/B_, serves an important anchoring function that may facilitate SNARE's modulation of Ca_V_2.1 and Ca_V_2.2 gating (Sheng et al. 1994, 1997; Rettig et al. 1996; Kim and Catterall 1997; Jarvis et al. 2002). Nonetheless, functional studies also suggest that the regulatory action of SNAREs might involve binding to other sites in the pore‐forming *α*_1_ channel subunit, and LI‐II and the C‐tail regions have been proposed as candidates (Bezprozvanny et al. 2000; Serra et al. 2010). Supporting this idea, recent findings show that low voltage‐activated Ca_V_3.x (T‐type) *α*_1_ channel subunits, which do not contain the consensus *synprint* site, biochemically interact with syntaxin‐1A and SNAP‐25 at the carboxy‐terminal domain (Weiss et al. 2012). In particular, syntaxin‐1A binding to Ca_V_3.x channels potently modulates channel gating in a similar way that found for Ca_V_2.x channels (Weiss et al. 2012). Besides, Ca_V_3.x‐SNAREs interaction also appears essential for T‐type channel‐triggered low‐threshold exocytosis (Weiss et al. 2012), thus providing a molecular mechanism for their coupling to neurotransmitter and hormone release in neurons and neuroendocrine cells near resting conditions or during mild stimulations (Carbone et al. 2014).

In conclusion, our data suggest that conformational modifications of *α*_1A_ LI‐II (due to the binding of a particular regulatory *β* subunit, mutation A454T (Serra et al. 2010), or deletion ΔLI‐II_451–457_) determine the modulation of Ca_V_2.1 steady‐state inactivation either by Ca^2+^ or by SNAREs but not by both.

## Conflict of Interest

The authors declare that no conflict of interests exists.

## In memoriam

In memory of Gemma G. Genè, PhD (1977–2017).
